# Bedarfsanalyse zur Curriculumsentwicklung für die anästhesiologische Kreißsaalversorgung – eine deutschlandweite Umfrage

**DOI:** 10.1007/s00101-022-01172-1

**Published:** 2022-07-13

**Authors:** Markus Flentje, Hendrik Eismann, Simon Schwill, Daniel Forstner, Peter Kranke

**Affiliations:** 1 Klinik für Anästhesiologie und Intensivmedizin, Medizinische Hochschule Hannover, Carl-Neuberg-Str. 1, 30625 Hannover, Deutschland; 2 Abteilung Allgemeinmedizin und Versorgungsforschung, Universitätsklinikum Heidelberg, INF 130.3, 69120 Heidelberg, Deutschland; 3 Gäubodenkaserne, SanLehrRgt Niederbayern, Mitterharthausen 55, 94351 Niederbayern, Deutschland; 4 Klinik und Poliklinik für Anästhesiologie, Intensivmedizin, Notfallmedizin und Schmerztherapie, Oberdürrbacher Str. 6, 97080 Würzburg, Deutschland

**Keywords:** Analgesie, Schnittentbindung, Weiterbildung, Geburt, Evaluation, Analgesia, Cesarean section, Further education, Birth, Evaluation

## Abstract

**Hintergrund:**

Die anästhesiologische Tätigkeit in der Kreißsaalumgebung impliziert die Besonderheiten der 200 %-Letalität, die beschreibt, dass Notfallsituationen Mutter und Kind betreffen können. Ein Umstand, der die Notwendigkeit einer besonderen Sorgfalt in der Mitarbeiterausbildung und -auswahl eindrücklich unterstreicht. Gleichwohl existiert derzeit keine detaillierte Beschreibung der notwendigen Kompetenzen in dieser Arbeitsumgebung. Die vorliegende Studie soll die Ausbildungssituation in der anästhesiologischen Weiterbildung beschreiben und im Hinblick auf die Notwendigkeit eines Curriculums analysieren.

**Methodik:**

In einer multizentrischen Beobachtungsstudie wurden Ärzt:innen in Weiterbildung (AiW) und ausbildende Fachärzt:innen (FÄ) nach Methoden der Einarbeitung, Feedbackgabe, übernommenen Tätigkeiten und Bedarf eines Curriculums befragt. Teilnehmende wurden über die Mitgliedsdatenbank der DGAI angeschrieben und konnten onlinebasiert den 11 Items umfassenden Fragebogen beantworten.

**Ergebnisse:**

Insgesamt wurden 495 Fragebogen (FÄ 329;166 AiW) abgeschlossen. Fachärzt:innen und AiW machen unterschiedliche Angaben zur Durchführung von Abschlussgesprächen (59,6 % vs. 10 %) und der Unterstützung durch ein Curriculum (76,3 % vs. 15,7 %). Unabhängig vom Weiterbildungsjahr werden von AiW Kaiserschnitte unter Supervisionsstufe „Rufweite“ durchgeführt. Die Periduralanästhesie (PDA) ist während der Einarbeitung die am seltensten durchgeführte Maßnahme. Beide Gruppen schätzen den Nutzen bzw. den Wert einer Beschreibung von Lernzielen und der Verfügbarkeit eines Curriculums als hoch ein.

**Schlussfolgerung:**

Die Unterstützung der Einarbeitung wird von FÄ und AiW teilweise unterschiedlich beantwortet. Einzelne seltene durchgeführte Maßnahmen, wie die PDA, bedürfen künftig einer gesonderten Aufmerksamkeit. Die Beschreibung von Lernzielen und die Curriculumsentwicklung werden ausdrücklich gewünscht.

**Zusatzmaterial online:**

Die Online-Version dieses Beitrags (10.1007/s00101-022-01172-1) enthält den Fragebogen zur Arbeit.

## Abstract (en)

**Background:**

Anesthesiologic activity in the delivery room environment implies the specifics of a 200% lethality, which describes that emergency situations can affect mother and child. A circumstance that impressively underlines the need for special care in employee training and selection. The training situation in the delivery room is characterized by several difficulties. Technical procedures are often performed on the awake patient, who is herself in an exciting situation during childbirth. A detailed description of the necessary competences in this working environment does not exist at the present. The present study aims to describe the further education situation in anesthesiology. The results can represent the first step of a curriculum development according to the concept of Kern et al. in the sense of a needs assessment.

**Material and methods:**

In a multicenter observational study, doctors in further training (AiW) and consultants (FÄ) were asked about methods of familiarization, feedback, activities taken on and the need for a curriculum. The level of supervision and confidence in action during procedures was also elicited. Participants were contacted via the membership database of the German Society of Anesthesia and Intensive Care and could answer the 11-item questionnaire online.

**Results:**

A total of 495 questionnaires (329 FÄ; 166 AiW;) were completed. The FÄ and AiW gave different information on the conduct of exit interviews (59.6% vs. 10%) and curriculum support (76.3% vs.17.5%). Independent of the year of training, AiWs perform cesarean sections under on-demand (reactive) supervision. Peridural anesthesia (PDA) is the least frequently performed procedure in the context of the familiarization situation with obstetric anesthesia. The groups have a different confidence of security in the successful implementation of procedures, when AiW are proceeding without direct supervision (FÄ = MD 61; AiW = MD 77; *p* < 0.001; scale 0 = unsecure–100 = very secure). Practical and technical support is mostly provided immediately by FÄ (> MW 91; scale 0 = never–100 = immediately). Individual values deviate significantly from the average values (outliers). Both groups rate the usefulness or value of describing learning objectives and the availability of a curriculum as high.

**Discussion:**

The support of familiarization and continuing support is partly answered differently by consultants and doctors in further training. Individual procedures that are rarely performed, such as administration of a PDA, require special attention in the future. Curricula with workplace-based assessments could provide feedback and assurance to increase confidence in the successful implementation of procedures by AiW. The AiW usually receive immediate professional support. Individual institutions do not seem to have any concepts here and must rethink their processes. This fact is particularly important against the background of the medicolegal significance of the subject. The description of learning objectives and curriculum development is desired both by FÄ and AiW.

## Einleitung

Die Anästhesiologie definiert sich als Fachgebiet über die 4 Säulen Anästhesie, Intensiv‑, Notfall- und Schmerzmedizin. Diese Aufgabengebiete werden teilweise auch von anderen Fachbereichen ausgeübt, und weitere Versorgungsschwerpunkte, wie z. B. die Palliativmedizin, gehören vielerorts jenseits dieser traditionellen Säulen zum anästhesiologischen Tagesgeschäft [1]. Technische Weiterentwicklungen in jedem dieser Teilbereiche und die Versorgung spezieller Patientengruppen werfen die grundsätzlichen Fragen auf, welche Kompetenzen in der Weiterbildung Anästhesi(ologi)e sicher erlangt werden sollten. Darüber hinaus kann es ergänzende Darstellungen von Kompetenzen geben. Die Empfehlungen zur Anwendung der Sonographie [2], die Betreuung kardiochirurgischer bzw. pädiatrischer oder gar die Versorgung von pädiatrisch-kardiochirurgischen Patienten [3] sind Beispiele für die sich zusehends entwickelnden speziellen Tätigkeiten.

Die Geburtshilfe/der Kreißsaal stellt ebenfalls einen speziellen Einsatzort in der Anästhesie dar. Umgangssprachlich hat sich der Begriff der 200%igen Letalität etabliert, der beschreibt, dass sich in Notfallsituationen Mutter und Kind in einer bedrohlichen Situation befinden. Das Spektrum kann – je nach Versorgungsstufe bzw. organisationaler Aufstellung – von der Versorgung eines unreifen Neugeborenen bis zur Behandlung schwerwiegender peri- oder postpartaler Blutungen reichen.

Für die Aus‑, Weiter- und Fortbildung des anästhesiologischen Personals im Kreißsaal bestehen verschiedenste Schulungsansätze, deren Themenschwerpunkte beispielsweise auf der allgemeinen geburtshilflichen Versorgung im interprofessionellen Team [4] oder der Versorgung spezieller Situationen, wie dem Notfallkaiserschnitt, liegen [5, 6]. Eine Empfehlung oder gar Pflicht zur Durchführung eines speziellen Programms existiert nicht. Richten sich diese Angebote an bereits im Kreißsaal tätige Mitarbeiter:innen (Ärzt:innen in Weiterbildung [ÄiW], Fachärzt:innen [FÄ]), gibt die Musterweiterbildungsordnung der Bundesärztekammer Orientierung für die Ausgestaltung vergleichbarer Schulungsangebote [7]. Zudem sollten die Inhalte nach Möglichkeit auf den Punkten des Nationalen Lernzielkatalogs „Anästhesiologie“ für Studierende aufbauen, deren Fortschreibung sie dann darstellen (Tab. 1; [8]). Das Bestehen vorhandener Ausbildungsbeschreibungen befreit nicht von der Evaluation und anschließenden Anpassung der Curricula. So wird zunehmend die Umstellung von reinen Zahlen- und Zeitvorgaben hin zu kompetenzbasierten Inhalten der ärztlichen Aus- und Weiterbildung empfohlen, welches neue Herausforderungen nach sich zieht [9, 10].

**Table 1:** 

Anästhesiologische Inhalte für die Kreißsaalumgebung aus der Musterweiterbildungsordnung [7]	Anästhesiologische Inhalte für die Kreißsaalumgebung des Nationalen Lernzielkatalog „Anästhesiologie“ [8]
Durchführung von Allgemeinanästhesien, Regionalanästhesien und perioperativer Behandlung bei Schwangeren	Anästhesierelevante anatomische und physiologische Besonderheiten der Schwangeren erklären
Schmerztherapie in der Geburtshilfe, einschließlich Kaiserschnitten	Die rechtlichen Besonderheiten bei der Aufklärung zur Anästhesie für geburtshilfliche Interventionen (Periduralanästhesie (PDA), Spinalanästhesie (SPA); Sectio) nennen
Durchführung von Anästhesieverfahren in der Geburtshilfe (50 Stück), davon bei Kaiserschnitten 25 Stück	Die Indikation präoperativer Gerinnungsdiagnostik für geburtshilfliche Regionalanästhesieverfahren kritisch diskutieren
–	Die Bedeutung der uteroplazentaren Perfusion durch medikamentöse Einflussgrößen erklären
–	Die Besonderheiten präoperativer Nüchternheit bei Schwangeren erklären
–	Den Begriff „walking epidural“ erklären
–	Lebensbedrohliche Komplikationen benennen (atonische Nachblutung, Fruchtwasserembolie, HELLP-Syndrom)
–	Geeignete Anästhesieverfahren zur Sectio caesarea benennen und in ihrem besonderen Risiko für die Schwangere erläutern

Um ein Curriculum sinnhaft und effektiv zu gestalten und einen hohen Akzeptanzgrad zu erzielen, sollte es strukturiert erstellt werden. Eines der bekanntesten Entwicklungsrahmen für eine strukturierte Herangehensweise bietet der Kern-Zyklus [11]. In 6 Schritten wird über Problemidentifikation, Vorerfahrungen, Zieldefinition, Ausbildungsstrategien, Implementierung bis zu Evaluation und Feedback ein Zyklus durchlaufen.

Ziel der vorliegenden Arbeit war es, im Sinne des Kern-Prozesses die aktuellen Rahmenbedingungen der Tätigkeiten von ÄiW im Kreißsaal zu beschreiben. Die Evaluation der bestehenden Einarbeitungssituation und die subjektive Beurteilung eines Bedarfs an weiteren Strukturen kann dann die Grundlage für die Entwicklung eines Kreißsaalcurriculums in diesem besonderen interdisziplinären wie interprofessionellen Setting darstellen.

## Methodik

Die Studie wurde als Querschnittsstudie durchgeführt. Zur Beschreibung der Einarbeitung und zu den Rahmenbedingungen wurde ein Fragenkatalog entwickelt, der sich an ÄiW und im Kreißsaal verantwortliche FÄ richtet. Die Befragung wurde über die Fachgesellschaft DGAI (Deutsche Gesellschaft für Anästhesiologie und Intensivmedizin) im Bundesgebiet unter den Mitgliedern als Onlineversion verbreitet. Die Studie wurde von der Ethikkommission der Medizinischen Hochschule und vom Datenschutzbeauftragen genehmigt (9603_BO_K_2021).

### Fragebogen

Anhand einer Literaturrecherche und den Vorerfahrungen der Autor:innen mit dem Setting (Kreißsaal) und der Aus‑, Weiter- und Fortbildungsbedingungen in der Anästhesie wurde ein zweigeteilter Fragebogen entwickelt. Ein Review fand im Arbeitsumfeld der Autoren durch FÄ Anästhesie statt und führte zur geringfügigen Anpassung. Mit insgesamt 12 Items sollten die Einarbeitung, fachliche Unterstützung und subjektiv empfundene Handlungskompetenz der Beteiligten erhoben werden. Als demografische Daten wurden Geschlecht und aktuelle Rolle in der Kreißsaalversorgung erfasst. Die Analyse nach überwachendem und anleitendem FÄ oder ÄiW mit jeweiligem Ausbildungsjahr war möglich. Anschließend wurden die Teilnehmer:innen (TN) innerhalb des Fragebogens zweizügig aufgeteilt, wobei einerseits die nachfolgenden Fragen aus Sicht eines anleitenden Mitarbeitenden (FÄ) und andererseits aus der Perspektive von ÄiW (1. bis 5. Jahr) gestellt werden konnten. Nach Abfrage der methodischen Unterstützung im Rahmen der Einarbeitung wurden die Tätigkeiten, Unterstützung bei den Tätigkeiten und die potenziellen Auswirkungen eines Curriculums erfragt. Die Intensität der Supervision von Maßnahmen wurde in die 2 Stufen „unter Sichtkontrolle“ und „in Rufweite“ eingeteilt. Es kamen 2 Fragetypen zum Einsatz. Geschlossene Fragen konnten mit Ja/Nein beantwortet werden. Aussagen und Items konnten von den TN durch Verstellen eines Schiebereglers bewertet werden, ohne die hinterlegte Skala von 0–100 (0: Ablehnung; 100: Zustimmung) zu sehen. Der vollständige Fragebogen ist im Zusatzmaterial online des Artikels verfügbar.

### Befragung

Nach Vorstellung der Studie im Wissenschaftlichen Arbeitskreis Geburtshilfliche Anästhesie der DGAI wurde die Umfrage über den E‑Mail-Verteiler an alle Mitglieder der DGAI disseminiert. Eine Erinnerung-E-Mail wurde 14 Tage nach dem initialen Aufruf geschickt.

Die Befragung wurde mittels eines onlinebasierten Fragebogens über die Umfragesoftware SurveyMonkey (Fa. SurveyMonkey Europe UC, Dublin, Irland) durchgeführt. Die Befragten erhielten einen Link zum Fragebogen. Nach Vorgaben des Datenschutzes wurden die IP-Adressen der TN nicht registriert. Die Befragung wurde vom 03.09.2019 bis zum 20.11.2019 durchgeführt.

### Statistik

Die erhobenen Daten wurden mithilfe von SPSS 26 (Fa. IBM Corporation, Armonk, NY, USA) ausgewertet. Die Ergebnisse der deskriptiven Statistik wurden als Median (*MD*), Interquartilabstand (IQR) und im Boxplot dargestellt. Um Unterschiede in den Gruppen zu beschreiben, wurde ein Mann-Whitney‑U Test durchgeführt.

## Ergebnisse

Insgesamt wurde der Link zum Fragebogen 1109-mal aufgerufen. 495 Fragebogen wurden abgeschlossen und gingen in die Bewertung ein. *N* = 329 der TN waren FÄ, *n* = 166 TN waren ÄiW. Die Verteilungen der verschiedenen Ausbildungsstufen und Rollen sind in Tab. 2 aufgeführt.

**Table 2:** 

**Mit welchen Methoden wurden Sie bei der Einarbeitung unterstützt?**						
*TN*	*Eingangsgespräch*	*Zwischengespräche*	*Abschlussgespräch*	*Einarbeitungsskript*	*Einarbeitungscurriculum*	*Keine Unterstützung*
ÄiW-gesamt *n* = 166	68 % *n* = 113	30 % *n* = 50	11 % *n* = 18	17 % *n* = 29	16 % *n* = 26	19 % *n* = 32
ÄiW 1. Jahr *n* = 8	75 % *n* = 6	50 % *n* = 4	0 % *n* = 0	25 % *n* = 2	38 % *n* = 3	0 % *n* = 0
ÄiW 2. Jahr *n* = 22	82 % *n* = 18	27 % *n* = 6	14 % *n* = 3	18 % *n* = 4	14 % *n* = 3	9 % *n* = 2
ÄiW 3. Jahr *n* = 25	76 % *n* = 19	44 % *n* = 11	16 % *n* = 4	28 % *n* = 7	12 % *n* = 3	12 % *n* = 3
ÄiW 4. Jahr *n* = 35	63 % *n* = 22	26 % *n* = 9	0 % *n* = 0	9 % *n* = 3	26 % *n* = 9	20 % *n* = 7
ÄiW 5. Jahr *n* = 76	63 % *n* = 48	26 % *n* = 20	14 % *n* = 11	17 % *n* = 13	11 % *n* = 8	26 % *n* = 20
**Mit welchen Methoden haben Sie die Einarbeitung unterstützt?**						
FA *n* = 329	85 % *n* = 179	65 % *n* = 214	40 % *n* = 133	30 % *n* = 97	24 % *n* = 78	4 % *n* = 14

### Didaktische Unterstützung und Einarbeitung

Tab. 2 zeigt die unterstützenden Maßnahmen, die den ÄiW zur Hilfe in der Weiterbildung angeboten werden. Unterschiede gibt es hinsichtlich der benannten Methoden zwischen der Nennung durch ÄiW und FÄ. So geben beispielsweise 15,7 % der ÄiW an, durch ein Curriculum unterstützt zu werden, 76,3 % der befragten FÄ geben an, ein solches zur Verfügung zu stellen. Auch die Antworten auf die Frage zu durchgeführten Abschlussgesprächen weisen erhebliche Unterschiede auf. Führen 59,6 % der FÄ ein solches Gespräch durch, geben nur 10 % der ÄiW an, ein solches Gespräch zu erhalten.

### Durchführung von Maßnahmen

Die Tätigkeiten der ÄiW unter Supervision sind in Tab. 3. dargestellt. Die ÄiW in den ersten beiden Weiterbildungsjahren nehmen zu einem größeren Anteil an der Aufklärungsarbeit für Anästhesieverfahren teil, als jene Maßnahmen selber durchgeführt werden. Die Anlage von Periduralkathetern (PDK) weist sowohl unter Supervision „unter Sichtkontrolle“ als auch unter Supervision „in Rufweite“ von FÄ die geringsten Werte auf (22 %/37 %). Ein Großteil der TN begleitet unabhängig vom Weiterbildungsjahr Kaiserschnitte mit der Supervisionsstufe „FÄ in Rufweite“ und nicht mit direkter Präsenz, also mit Supervision „unter Sichtkontrolle“.

**Table 3:** 

TN der Umfrage	Aufklärung von Patientinnen zum Kaiserschnitt	Aufklärung von Patientinnen zur PDK-Anlage	Anlage von PDK (Supervision, Sichtkontakt)	Anlage von PDK (FA in Rufweite)	Betreuung, Eingriff Sectio (Supervision, Sichtkontakt)	Betreuung, Eingriff Sectio (Supervision Rufweite)	Keine
ÄIW-gesamt (*n* = 166)	83 % *n* = 137	95 % *n* = 157	22 % *n* = 37	37 % *n* = 61	33 % *n* = 54	76 % *n* = 126	1 % *n* = 1
ÄIW 1. Jahr (*n* = 8)	88 % *n* = 7	88 % *n* = 7	38 % *n* = 3	50 % *n* = 4	38 % *n* = 3	88 % *n* = 7	0 *n* = 0
ÄIW 2. Jahr (*n* = 22)	86 % *n* = 19	100 % *n* = 22	41 % *n* = 9	50 % *n* = 11	45 % *n* = 10	68 % *n* = 15	0 % *n* = 0
ÄIW 3. Jahr (*n* = 25)	84 % *n* = 21	96 % *n* = 24	28 % *n* = 7	40 % *n* = 10	40 % *n* = 10	72 % *n* = 18	0 % *n* = 0
ÄIW 4. Jahr (*n* = 35)	77 % *n* = 27	97 % *n* = 34	14 % *n* = 5	31 % *n* = 11	43 % *n* = 15	89 % *n* = 31	0 % *n* = 0
ÄIW 5. Jahr (*n* = 76)	83 % *n* = 63	92 % *n* = 70	17 % *n* = 13	33 % *n* = 25	21 % *n* = 16	89 % *n* = 55	1 % *n* = 1
*Welche Maßnahmen führen die von Ihnen betreuten ÄIW durch?*							–
FA *n* = 329	88 % *n* = 291	93 % *n* = 306	88 % *n* = 288	79 % *n* = 261	92 % *n* = 304	80 % *n* = 179	1 % *n* = 4

*FA* Fachärtz:innen, *ÄiW* Ärztin/Arzt in Weiterbildung, *PDK* Periduralkatheter, *TN* Teilnehmer:in

### Unterstützung durch Fachärzt:innen

**Figure 1:**
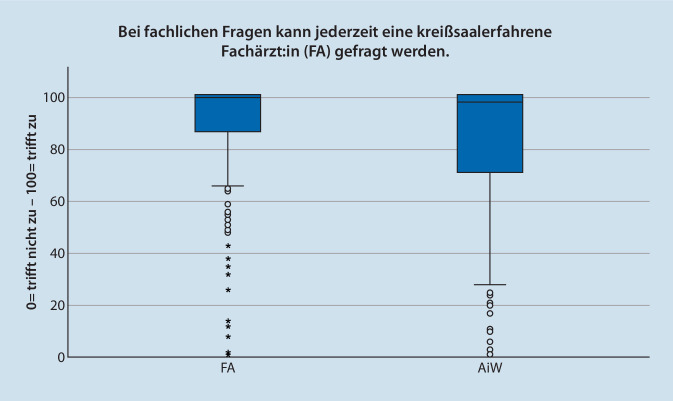

Die Frage nach sofortiger Unterstützung durch FÄ bei Fragen wurden von den überwachenden FÄ und ÄiW unterschiedlich beantwortet. Die Bewertung nach theoretisch fachlicher Unterstützung wurde von FÄ mit MD = 100 (*n* = 296; IQR = 15; Max = 100; Min = 0) und von ÄiW mit MD = 98 (*n* = 163; IQR = 30; Max = 100; Min = 0) beantwortet. Die Frage nach sofortiger praktischer Unterstützung wurde von FÄ mit MD = 100 (*n* = 296; IQR = 18; Max = 100; Min = 0) und von ÄiW mit MD = 91 (*n* = 163; IQR = 36; Max = 100; Min = 0) signifikant niedriger bewertet (*p* < 0,001). In Abb. 1 und 2 sind die Daten im Boxplot dargestellt.

**Figure 2:**
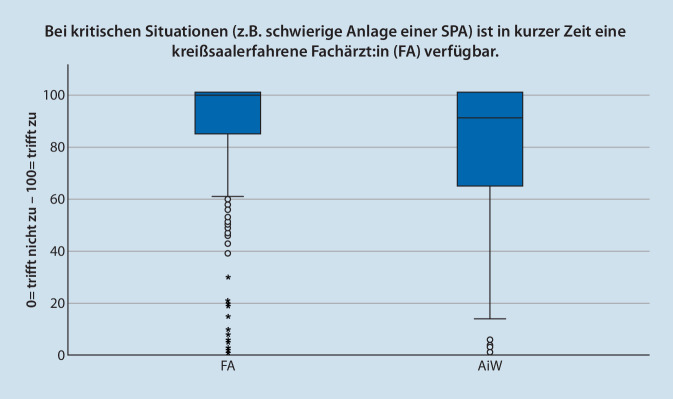

### Subjektives Sicherheitsgefühl

**Figure 3:**
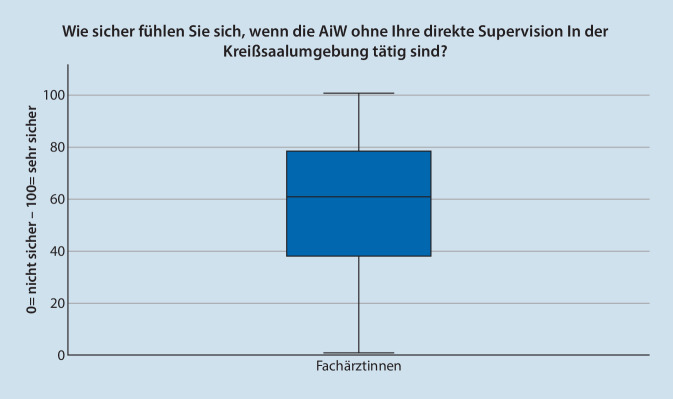

**Figure 4:**
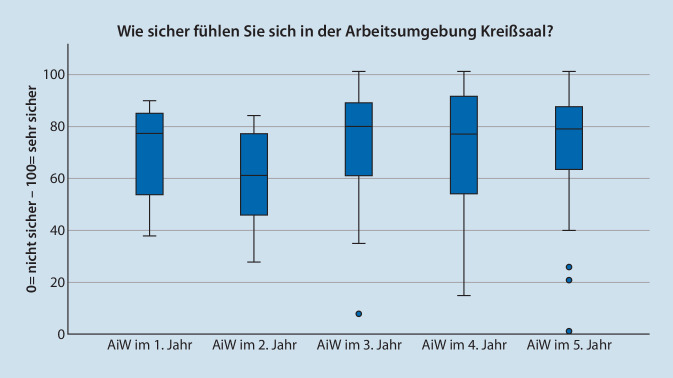

Die FÄ bewerteten das subjektive Sicherheitsgefühl mit MD = 61 (*n* = 296; IQR = 40; Max = 100; Min = 0), wenn ÄiW ohne direkten Sichtkontakt tätig sind (Abb. 3). Die ÄiW bewerten ihr eigenes Sicherheitsgefühlt mit MD = 77 (*n* = 166; IQR = 29; Max = 100; Min = 0). Die Werte sind signifikant unterschiedlich (*p* < 0,001). Abb. 4 zeigt die Werte nach Weiterbildungsjahr aufgeteilt.

### Bewertung der Einarbeitung und Curriculumsbedarf

**Figure 5:**
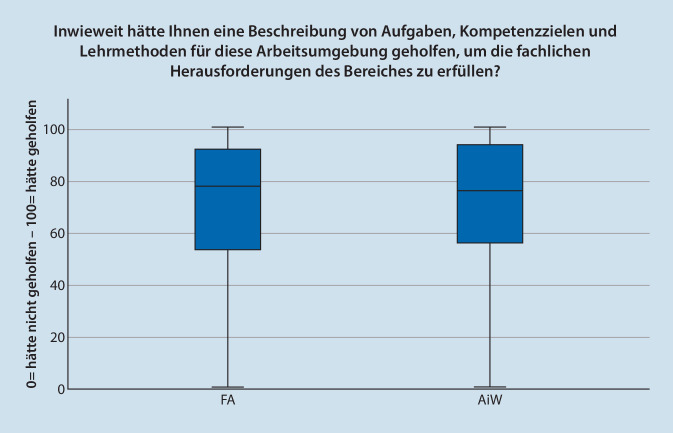

Insgesamt wird die Suffizienz der Einarbeitung in die Arbeitsumgebung Kreißsaal von FÄ mit *MD* = 71 (*n* = 325; IQR = 33; Max = 100; Min = 0) bewertet. Die Einschätzung der ÄIW ist mit *MD* = 52 (*n* = 163; IQR = 45, Max = 100; Min = 0) signifikant niedriger (*p* < 0,001). Der Nutzen einer Beschreibung von Lernzielen und eines Curriculums wird von den Befragten mit der Rolle FA mit MD = 78 (*n* = 296; IQR = 39; Max = 100; Min = 0) bzw. MD 81(*n* = 296; IQR = 42; Max = 100; Min = 0) bewertet. In der Rolle ÄiW erreichten die Werte MD = 77 (*n* = 163; IQR = 38; Max = 100; Min = 0) und MD = 79 (*n* = 163; IQR = 42; Max = 100; Min = 0) eingeordnet (Abb. 5 und 6). Einen signifikanten Unterschied zwischen den Rollen ist nicht vorhanden.

**Figure 6:**
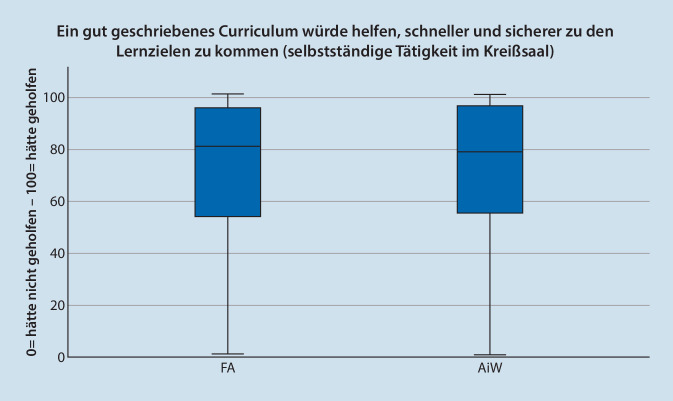

## Diskussion

Ziel der vorliegenden Arbeit war es, die aktuelle Einarbeitungssituation der ÄIW und den Bedarf für die Erstellung eines Kreißsaalcurriculums in der Facharztweiterbildung Anästhesie zu beschreiben.

In der vorliegenden Studie konnten wir die Einarbeitungsmethoden und aktuellen Tätigkeiten der ÄiW in die Kreißsaalumgebung beschreiben, die im Verlauf selbstständiger wird und am häufigsten durch die Methode des Eingangsgespräches begleitet wird. Abschlussgespräche finden aus Sicht der ÄiW am seltensten statt. Die PDA ist die am seltensten durchgeführte Maßnahme, obwohl sie im Rahmen der geburtshilflichen Anästhesie sicherlich zu den Kernkompetenzen zu zählen ist. Die gefühlte Sicherheit zwischen FÄ und ÄiW unterscheidet sich und ist beim ÄiW höher. Curricula mit arbeitsplatzbasierten Assessments könnten hier Feedback und Sicherheit geben. Die Erstellung eigener detaillierter Beschreibungen der Kompetenzen im Kreißsaal und eines dafür ausgerichteten Curriculums mit dem Fokus der anästhesiologischen Kreißsaalumgebung wird von den Zielgruppen als ausgesprochen wünschenswert angesehen.

Die Ausbildungssituation in der Kreißsaalumgebung ist durch mehrere Hürden und Schwierigkeiten charakterisiert. Technische Maßnahmen werden oft an der wachen Patientin durchgeführt, die sich selbst unter der Geburt in einer Extremsituation befindet. Dennoch müssen ÄiW während der oftmals kurzen Ausbildungsphase (Stichwort: „*bottleneck“*) in dieser Arbeitsumgebung mit dem Ziel eingesetzt werden, rasch eine hohe Handlungskompetenz zu erreichen. Kompetenzen entwickeln sich schrittweise und werden von Ten Cate et al. in einer Beziehung zwischen Ausbildenden und Lernenden anhand eines abnehmenden Grades der notwendigen Supervision beschrieben [12]. Angelehnt an dieses Modell kann bei Übertragung auf die anästhesiologische Kreißsaaleinarbeitung nicht von einer „Ad-hoc“-Kompetenz ausgegangen werden, sondern es ist vielmehr ein „Hereinwachsen“ in die Rolle mit zunehmend geringer werdendem Supervisionsbedarf notwendig.

In welcher Phase und Art Mitarbeiter:innen in dieser Arbeitsumgebung tätig werden, kann von lokalen strukturellen Gegebenheiten und von dem zu versorgenden Patientenkollektiv beeinflusst werden. So führen sowohl ÄiW im ersten Weiterbildungsjahr schon die anästhesiologische Betreuung von Kaiserschnitten unter Supervisionsstufe „FÄ in Rufweite“ durch, andererseits haben nicht alle ÄiW im 5. Jahr diese Stufe erreicht. Fraglich ist, inwieweit der Rückgang der Geburtskliniken in Deutschland darüber hinaus in diesem Aspekt eine Rolle spielt. So ist seit 1991 die Anzahl von Geburtskliniken um 40 % zurückgegangen [13]. Denkbare wäre, dass, einhergehend mit diesem Rückgang, ÄiW an in ihrer Arbeitsumgebung weniger direkten Kontakt zur Geburtshilfe haben und die geforderten Leistungen in Rotationen in anderen Kooperationskliniken erreichen müssen. Zahlen existieren hier nach Kenntnis der Autoren nicht.

Die Angaben von FÄ und ÄiW variieren bei einigen Items zu den Fragen der Einarbeitung und der Maßnahmen erheblich. Diese Disparität kann ihre Begründung in einer unterschiedlichen Herkunft der teilnehmenden ÄiW und FÄ haben. Darüber hinaus neigen TN dazu, an der Umfrage teilzunehmen, wenn sie diese selber als wichtig interpretieren. Beispiele zur Teilnahmemotivation wären ein Mangel oder eine verbesserungswürdige Situation oder umgekehrt Lehrende, die stolz auf Errungenschaften in Bezug auf ein erarbeitetes Einarbeitungskonzept sind [14, 15]. Dieser Bias ist gleichwohl im Hinblick auf den evaluierten Bedarf eines Curriculums nicht ausschlaggebend.

Mit 15,7 % der TN aus der Gruppe der ÄiW ist der Anteil der TN, die bereits über ein Curriculum verfügen, so klein, dass die Annahme berechtigt erscheint, dass derzeit eine große Anzahl ÄIW ohne Curriculum eingearbeitet wird. Bei den zur Anwendung kommenden Methoden dominiert das Einarbeitungsgespräch. Zwischen- und Abschlussgespräche finden in deutlich geringerer Zahl statt. Vor dem Hintergrund, dass ein gut durchgeführtes Feedback einer der stärksten Faktoren ist, lernen zu beeinflussen [16], wird diese Möglichkeit überraschend wenig genutzt.

Die Anlage der Periduralanästhesie (PDA) erreicht in der Umfrage als durchgeführte Maßnahme die geringsten Werte (22–37 %). Die Autoren interpretieren dies damit, dass die PDA eher ungeplant angefordert wird. Eine zu oft erfolgende Verschiebung dieser Maßnahme in den Bereitschaftsdienst und damit zu selten auftretende Möglichkeiten der Anlage tagsüber bietet nach der Literatur kein Argument, da die Anforderung der PDA zeitunabhängig beschrieben wird [17]. Gleichwohl werden für diese Maßnahme im Ausbildungssetting auch zur Regelarbeitszeit 2 Mitarbeiter:innen (FÄ und ÄiW) gebunden und stehen damit dem Routinebetrieb nicht mehr zur Verfügung. Das Erlernen der PDA kann, hieraus folgend, ein Beispiel für die Notwendigkeit eines besonderen Ausbildungsfokus, z. B. unter Einsatz von Simulatoren, bieten.

Beantworten die FÄ die Frage nach sofortiger Möglichkeit der theoretischen und praktischen Unterstützung des ÄiW im Kreißsaal mit annähernd 100 %, liegt die Bewertung der ÄIW mit 98,5 % bzw. 91 % etwas niedriger. Problematisch scheinen in dieser Frage insbesondere die in den Abb. 1 und 2 dargestellten Ausreißer nach unten zu sein. Bei anderen Vorgaben aus dem deutschen anästhesiologischen Umfeld werden Umsetzungen von 70–80 % beschrieben [18, 19], sodass die einzelnen – niedrigen – Werte nicht weiter überraschen. Mit dem Anspruch der Patient:innen auf fachärztliche Betreuung muss die Überwachung der Tätigkeit von ÄiW durch Möglichkeiten des Einschreitens durch FÄ jederzeit garantiert werden [20].

Das Sicherheitsgefühl von FÄ bei Tätigkeiten von ÄiW ist geringer als das subjektive Gefühl von ÄiW. Da die Autoren den Wert mit 60 % (FÄ) als relativ niedrig einschätzen, wäre es interessant zu wissen, ob die FÄ durch organisatorische Rahmenbedingungen genötigt werden, keine direkte Überwachung durchführen zu können. Die Diskrepanz zwischen Lehrenden und Lernenden steht im Einklang mit der Literatur zu anderen anästhesiologischen Tätigkeiten [21]. Die Selbsteinschätzung von ÄiW kann durch den sog. Dunning-Krüger Effekt überlagert werden, welcher auch aus dem medizinischen Umfeld bekannt ist und beschreibt, dass es gerade bei weniger Kompetenz zur Überschätzung der Fähigkeiten kommt [22]. Darüber hinaus könnten strukturierte Anteile von arbeitsplatzbasiertem Assessment, wie diese z. B. im Konzept der „entrustable professional activities“ (EPA) vorgesehen sind, Lehrenden und Lernenden Sicherheit zum eigenen Kompetenzniveau in abgeschlossenen Arbeitseinheiten geben [23]. In Deutschland gibt es erste Konzepte, EPA fest in die anästhesiologische Ausbildung zu integrieren, wobei sich in einer ersten Analyse 2 EPA auf die Kreißsaalumgebung beziehen [24]. Damit ist die Anzahl von 2 EPA mit Kreißsaalbezug vergleichbar zu internationalen Arbeiten. Hier werden im Speziellen die Begleitung einer „Kaiserschnittoperation“ und das „geburtshilfliche Schmerzmanagement“ benannt [25]. Internationale Fortbildungskonzepte orientieren sich inhaltlich eher rückwärtsgerichtet an den am häufigsten entstandenen Schadensfällen [26].

Der Bedarf nach einer detaillierten Beschreibung von Tätigkeiten, Kompetenzzielen und Lehrmethoden und der Bedarf eines Curriculums wurden von FÄ und ÄiW insgesamt als hoch eingeschätzt. Diese Bewertung reflektiert die kulturellen Wandel der letzten Jahrzehnte in der medizinischen Aus- und Weiterbildung, der die Professionalisierung und Strukturierung dieses Bereiches zunehmend fordert [27]. Die Befragungsstudie zeigt, dass weitere Anstrengungen in die detaillierte Curriculumsentwicklung lohnenswert sind.

### Limitation

Der Fragebogen zur Bewertung der Arbeitsumgebung im Kreißsaal wurde von den Autoren entwickelt und bislang nicht in größerem Ausmaß validiert. Es ist möglich, dass weitere Items bestehen, die das Arbeitsumfeld abbilden. Die Methodik der onlinebasierten anonymen Umfrage führt zu Limitationen, die bei Interpretation der Studie beachtet werden sollten. Das Datenschutzgutachten ließ keine Dokumentation der IP-Adressen zu, sodass theoretisch ein mehrfaches Ausfüllen des Fragebogens möglich gewesen wäre, gleichwohl aber als unwahrscheinlich eingestuft wird. Die fehlende *Baseline* in Bezug auf anästhesiologische Mitarbeiter:innen mit direktem Kontakt zur Kreißsaalumgebung (Wer ist überhaupt in der Kreißsaaleinarbeitung tätig?) erlaubt keine Bewertung im Sinne einer Rücklaufquote.

## Fazit für die Praxis

- Methoden des Feedbacks, wie Zwischen- und Abschlussgespräche, können für die anästhesiologische Einarbeitung in die Kreißsaalumgebung häufiger genutzt werden.
- Im Einzelfall ist die akute Verfügbarkeit von anästhesiologischen FÄ in der Kreißsaalumgebung zu überprüfen.
- Die Erstellung eigener detaillierter Beschreibungen der Kompetenzen im Kreißsaal und eines dafür ausgerichteten Curriculums mit dem Fokus der anästhesiologischen Kreißsaalumgebung wird als wünschenswert angesehen.

## Supplementary Information




