# Supplementary material for: Analyses of requirements for curriculum development for the training of anesthesiologists in the delivery room—A nationwide survey
Source: Anaesthesiologie. 2022 Jul 13;71(9):697–705. [Article in German] doi: 10.1007/s00101-022-01172-1 (PMC9427909; doi:10.1007/s00101-022-01172-1)
Supplement: Supplementary file 1 [file 101_2022_1172_MOESM1_ESM.pdf]

Zusatzmaterial zum Beitrag „Bedarfsanalyse zur Curriculumsentwicklung für die anästhesiologische Kreißsaalversorgung. Eine deutschlandweite Umfrage“ von Flentje M, Eismann H, Schwill S et al. (2022) in *Die Anaesthesiologie*

Beitrag und Zusatzmaterial stehen Ihnen auf www.springermedizin.de zur Verfügung. Bitte geben Sie dort den Beitragstitel in die Suche ein.

Fragebogen

<table>
<tr><td colspan="2">Allgemeine Fragen</td></tr>
<tr><td colspan="2">Sie sind:
Männlich
Weiblich
Divers
Keine Angaben</td></tr>
<tr><td colspan="2">Wie ist Ihre derzeitige Rolle im Kreißsaal?
Überwachung und Begleitung auszubildender Weiterbildungsassistenten im Kreißsaal
Arzt/Ärztin in Weiterbildung im 1. Jahr
Arzt/Ärztin in Weiterbildung im 2. Jahr
Arzt/Ärztin in Weiterbildung im 3. Jahr
Arzt/Ärztin in Weiterbildung im 4. Jahr
Arzt/Ärztin in Weiterbildung im 5. Jahr</td></tr>
<tr><td>Fragen Fachärt:innen in anleitender Position</td><td>Fragen Ärzt:innen in Weiterbildung</td></tr>
<tr><td>Mit welchen Mitteln unterstützen Sie die die Einarbeitung der Weiterbildungsassistent*innen in Ihrem Arbeitsalltag?
• Eingangsgespräch
• Zwischengespräch
• Abschlussgespräch
• Einarbeitungsskript
• Einarbeitungscurriculum
• keine Unterstützung</td><td>Mit welchen Mitteln wurden Sie bei der Einarbeitung der in die Kreißsaalumgebung unterstützt?
• Eingangsgespräch
• Zwischengespräch
• Abschlussgespräch
• Einarbeitungsskript
• Einarbeitungscurriculum
keine Unterstützung</td></tr>
<tr><td>Welche Maßnahmen führen Ihre Weiterbildungsassistenten*innen im Kreißsaal durch? (Mehrfachantworten möglich)
• Aufklärung von Patient:innen für Kaiserschnitte
• Aufklärung von Patient:innen für die Anlage von Periduralkatheter
• Anlage von Periduralkatheter unter direkter Supervision (Sichtkontakt)</td><td>Welche Maßnahmen führen Sie im Kreißsaal durch? (Mehrfachantworten möglich)

• Aufklärung von Patient:innen für Kaiserschnitte
• Aufklärung von Patient:innen für die Anlage von Periduralkatheter
• Anlage von Periduralkatheter unter direkter Supervision (Sichtkontakt)</td></tr>
</table>

• Anlage von Periduralkatheter unter Supervision (Facharzt Rufweite, z.B. auf dem OP-Gang)<br>• Anlage von Periduralkathern ohne Supervision (Facharzt im Rufdienst)<br>• Betreuung von Patient:innen bei der Sectio (Anlage SPA etc.) unter direkter Supervision (Sichtkontakt)<br>• Betreuung von Patientinnen bei der Sectio (Anlage SPA etc.) unter Supervision (Facharzt Rufweite, z.B. auf dem OP-Gang)<br>• Betreuung von Patientinnen bei der Sectio (Anlage SPA etc.) unter Supervision (Facharzt Rufweite, z.B. auf dem OP-Gang)	• Anlage von Periduralkatheter unter Supervision (Facharzt Rufweite, z.B. auf dem OP-Gang)<br>• Anlage von Periduralkathern ohne Supervision (Facharzt im Rufdienst)<br>• Betreuung von Patient:innen bei der Sectio (Anlage SPA etc.) unter direkter Supervision (Sichtkontakt)<br>• Betreuung von Patientinnen bei der Sectio (Anlage SPA etc.) unter Supervision (Facharzt Rufweite, z.B. auf dem OP-Gang)
**Ratingfragen: Die Teilnehmer:innen bewerten die Aussage auf einem Schieberegler von links: trifft nicht zu bis rechts: trifft zu (Skala verborgen von 1= gering – 100 = sehr stark)**	
**Ärzt:nnen in Weiterbildung**	Wie suffizient bewerten Sie Ihre Einarbeitung in den Kreißsaal?
	Wie handlungssicher fühlen Sie sich bei den von Ihnen durchgeführten Maßnahmen im Kreißsaal?
	Bei fachlichen Fragen kann ich jederzeit einen Kreißsaal-erfahrenden Facharzt fragen
	Bei kritischen Situationen (z.B. schwierige Anlage einer SPA) ist in kurzer Zeit ein Kreißsaal-erfahrender Facharzt verfügbar.
**Fachärzt:innen**	Wie suffizient bewerten Sie Ihre Einarbeitung in den Kreißsaal?
	Wie ist Ihr persönliches Sicherheitsgefühl, wenn Weiterbildungsassistenten ohne Ihre direkte Supervision (Sichtkontakt) arbeiten?
	Bei fachlichen Fragen kann der AiW jederzeit einen Kreißsaal-erfahrenden Facharzt fragen
	Bei kritischen Situationen (z.B. schwierige Anlage einer SPA) ist in kurzer Zeit ein Kreißsaal-erfahrender Facharzt verfügbar.
**Die Teilnehmer:innen bewerten die Aussage auf einem Schieberegler von links: hätte nicht geholfen bis rechts: hätte geholfen (Skala verborgen von 1= gering – 100 = sehr stark)**	
**Ärzt:innen in Weiterbildung**	In wie weit hätte Ihnen eine Beschreibung von Aufgaben, Kompetenzzielen und Lehrmethoden für diese Arbeitsumgebung geholfen, um die fachliche Herausforderung des Bereiches zu erfüllen.

	Ein gut geschriebenes Curriculum würde mir helfen, schneller und sicherer zu meinem Lernziel (selbständige Tätigkeit im Kreißsaal) zu kommen.
**Fachärzt:innen**	In wie weit hätte Ihnen eine Beschreibung von Aufgaben, Kompetenzzielen und Lehrmethoden für diese Arbeitsumgebung geholfen, um die fachliche Herausforderung des Bereiches zu erfüllen.
	Ein gut geschriebenes Curriculum würde mir helfen, die AiW schneller und sicherer zu Ihrem Lernziel (selbständige Tätigkeit im Kreißsaal) zu bringen.

SPA=Supraspinale Anästhesie
